# Near-membrane ensemble elongation in the proline-rich LRP6 intracellular domain may explain the mysterious initiation of the Wnt signaling pathway

**DOI:** 10.1186/1471-2105-12-S13-S13

**Published:** 2011-11-30

**Authors:** Chengcheng Liu, Mingxi Yao, Christopher WV Hogue

**Affiliations:** 1 Computation and Systems Biology Programme, Singapore-MIT Alliance, E4-04-10, 4 Engineering Drive 3, Singapore; 2 Mechanobiology Institute Singapore, National University of Singapore, T-lab, #05-01, 5A Engineering Drive 1, Singapore; 3 Department of Biological Sciences, National University of Singapore, 14 Science Drive 4, Singapore

## Abstract

**Background:**

LRP6 is a membrane protein crucial in the initiation of canonical Wnt/β-catenin signalling. Its function is dependent on its proline-serine rich intracellular domain. LRP6 has five PPP(S/T)P motifs that are phosphorylated during activation, starting with the site closest to the membrane. Like all long proline rich regions, there is no stable 3D structure for this isolated, contiguous region.

**Results:**

In our study, we use a computational simulation tool to sample the conformational space of the LRP6 intracellular domain, under the spatial constraints imposed by (a) the membrane and (b) the close approach of the neighboring intracellular molecular complex, which is assembled on Frizzled when Wnt binds to both LRP6 and Frizzled on the opposite side of the membrane. We observe that an elongated form dominates in the LRP6 intracellular domain structure ensemble. This elongation could relieve conformational auto-inhibition of the PPP(S/T)PX(S/T) motif binding sites and allow GSK3 and CK1 to approach their phosphorylation sites, thereby activating LRP6 and the downstream pathway.

**Conclusions:**

We propose a model in which the conformation of the LRP6 intracellular domain is elongated before activation. This is based on the intrusion of the Frizzled complex into the ensemble space of the proline rich region of LRP6, which alters the shape of its available ensemble space. To test whether this observed ensemble conformational change is sequence dependent, we did a control simulation with a hypothetical sequence with 50% proline and 50% serine in alternating residues. We confirm that this ensemble neighbourhood-based conformational change is independent of sequence and conclude that it is likely found in all proline rich sequences. These observations help us understand the nature of proline rich regions which are both unstructured and which seem to evolve at a higher rate of mutation, while maintaining sequence composition.

## Background

**Figure 1:**
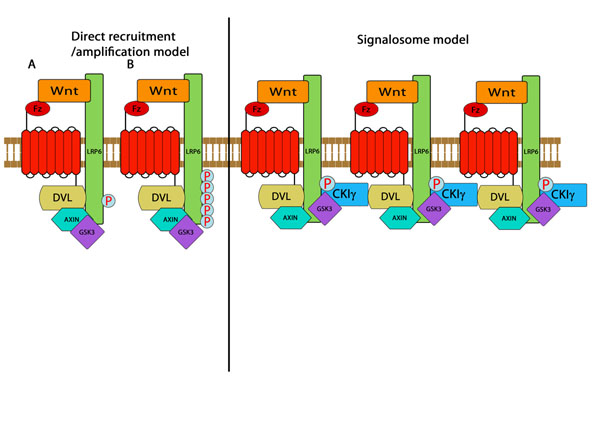
**Two proposed initiation models of canonical Wnt/β-catenin signalling pathways.** In the sequential recruitment/amplification model (left), Wnt-induced FZD-LRP6 complex formation promotes initial LRP6 phosphorylation via DVL recruitment of the AXIN-GSK3 complex. Initial LRP6 phosphorylation provides docking sites and thereby recruits additional AXIN-GSK3 complex to promote further LRP6 phosphorylation if LRP6 multimerizes. In the signalosome/aggregation model (right), Wnt induces clustering of LRP6, leading to its phosphorylation by CK1 and subsequently by GSK3 and recruitment of AXIN.

Wnt induced signaling pathways play essential roles in development and disease [1-3]. Currently, two initiation models of the canonical Wnt/β-catenin signaling pathway have been proposed as illustrated in Figure 1[4-6]. One could be referred to as the sequential recruitment/amplification model, in which Wnt stimulation is proposed to recruit the scaffold protein AXIN to approach the membrane through the bridging interactions between frizzled (FZD) and dishevelled (DVL), as well as between DVL and AXIN. GSK3 (glycogen synthase kinase 3) in association with AXIN thereafter is able to phosphorylate the LRP5/6 PPP(S/T)P motif near the membrane. Initial phosphorylation creates a docking site for AXIN and thereby recruits additional AXIN-GSK3 to promote further LRP6 phosphorylation [7]. The second model is the signalosome/aggregation model. Recent results showed that a signalosome is formed by aggregated LRP6 and AXIN when Wnt is present. Clustering of LRP6 leads to the phosphorylation of T1479 by CK1γ (casein kinase 1γ) and subsequent phosphorylation of the PPP(S/T)P motif by GSK3 [8]. Phosphorylated LRP6 recruits AXIN resulting in the disruption of the “β-catenin destruction complex”, which comprises AXIN, APC (the tumor suppressor *Adenomatous polyposis coli*), GSK3 and CK1α [9,10]. This results in the stabilization of a cytoplasmic pool of β-catenin. Free β-catenin enters the nucleus and activates gene transcription by binding to the TCF/LEF (T cell factor/Lymphoid enhancer factor) family of transcription factors [11-13]. Thereafter, the activation of LRP5/6 is indispensable to initiate the downstream intracellular Wnt signaling cascade, in order to stabilize β-catenin.

LRP6/LRP5/Arrow belongs to a subfamily of LDL receptors (LDLR) [14]. Human LRP6 is a type I single-pass transmembrane protein. Its modular extracellular domain has three basic domains; the YWTD (tyrosine, tryptopan, threonine and aspartic acid)-type β-propeller domain, the EGF (epidermal growth factor)-like domain, and the LDLR type A (LA) domain. This region has crystal structures present in PDB database [15-17]. During signaling pathway initiation, Wnt binds the cysteine-rich domain of FZD proteins and exhibits a Wnt1-dependent association with LRP6 extracellular domains *in vitro *[18,19]. However, the interaction between Wnt and LRP6 is weaker compared to the interaction between Wnt and Frizzled [18]. It is therefore more likely that a LRP6-FZD complex binds to the LRP6 extracellular domain. After deletion of its extracellular domain, LRP6 protein could still activate the Wnt/β-catenin signaling pathway [20].

The LRP6 intracellular domain is rich in prolines and serines. Sequence alignment shows that it includes a S/T cluster and downstream five reiterated PPP(S/T)PX(S/T) motifs, each of which contains a PPP(S/T)P motif phosphorylated by GSK3 and juxtaposed to a CK1 phosphorylation site [21]. Such dual phosphorylation is essential to stabilize the pool of β-catenin in the cytoplasm [22]. The phosphorylation of the S/T cluster has also been characterized, particularly the phosphorylation of T1479 by CK1γ [8,23]. It is believed that the phosphorylated S/T cluster promotes downward PPP(S/T)PX(S/T) phosphorylation [24]. He’s group previously showed that a LRP6 mutant lacking most of the intracellular domain is a loss-of-function [18]. In addition, a truncated LRP6 comprising its transmembrane and intracellular domain is constitutively active in Wnt signaling transduction [25-27]. A single PPP(S/T)P motif transferred to a LRP6 variant lacking the extracellular domain activates the Wnt/β-catenin signaling pathway. Phosphorylated PPP(S/T)PX(S/T) motifs provide docking sites for AXIN, which associates with GSK3 to promote proximity to LRP6 [28]. So far, no stable structure has been obtained from this isolated and contiguous LRP6 intracellular domain in current structure databases. The LRP6 intracellular domain is expected to be natively unstructured (unfolded or disordered) since its composition is enriched with proline whose conformation is limited [4,15,21,29]. There has been little study on the conformational behavior of LRP5/6 before activation, when Wnt induces signal transduction.

No matter which initiation model applies to the canonical Wnt/β-catenin signaling pathway, the conformation of LRP6 has to face spatial constraints imposed by (i) the plasma membrane and (ii) a nearby molecule or molecular assemblies, which could be neighboring LRP6 molecules or a Wnt-FZD-DVL-AXIN-GSK3 assembly. We hypothesize that these two spatial constraints would restrict LRP6 intracellular domain conformational space and so cause its conformation to adopt a more extended or elongated form before it is activated and docked by AXIN. We applied our TraDES software package [30] to simulate the conformational space of the LRP6 intracellular domain, as well as constraints to demonstrate an elongated conformational change of this domain occurs during Wnt/β-catenin signaling pathway initiation. We also tested whether close packing can induce a statistically observable structural change in an ensemble of unfolded states in a sequence independent manner.

## Results

### LRP6 intracellular domain is predicted to be unfolded

No stable structure has been documented for the LRP6 intracellular domain in current structure databases. This region is expected to be natively unstructured because it is enriched with proline and serine. Several protein disorder prediction tools predict that this region is disordered or unfolded. Figure S1 (in the Additional File 1) gives the prediction results from disorder predictors; RONN [31], IUPred [32], Globplot [33], PONDR-FIT [34] and FoldIndex [35]. This unfolded intracellular protein region most probably tends to have random coiled conformations, which auto-inhibits the structure itself to avoid interactions with other molecules [36]. Like most other disordered protein regions, it exists as an ensemble of structures which can be generated by TraDES in this simulation study.

### Radius of gyration distribution

**Figure 2:**
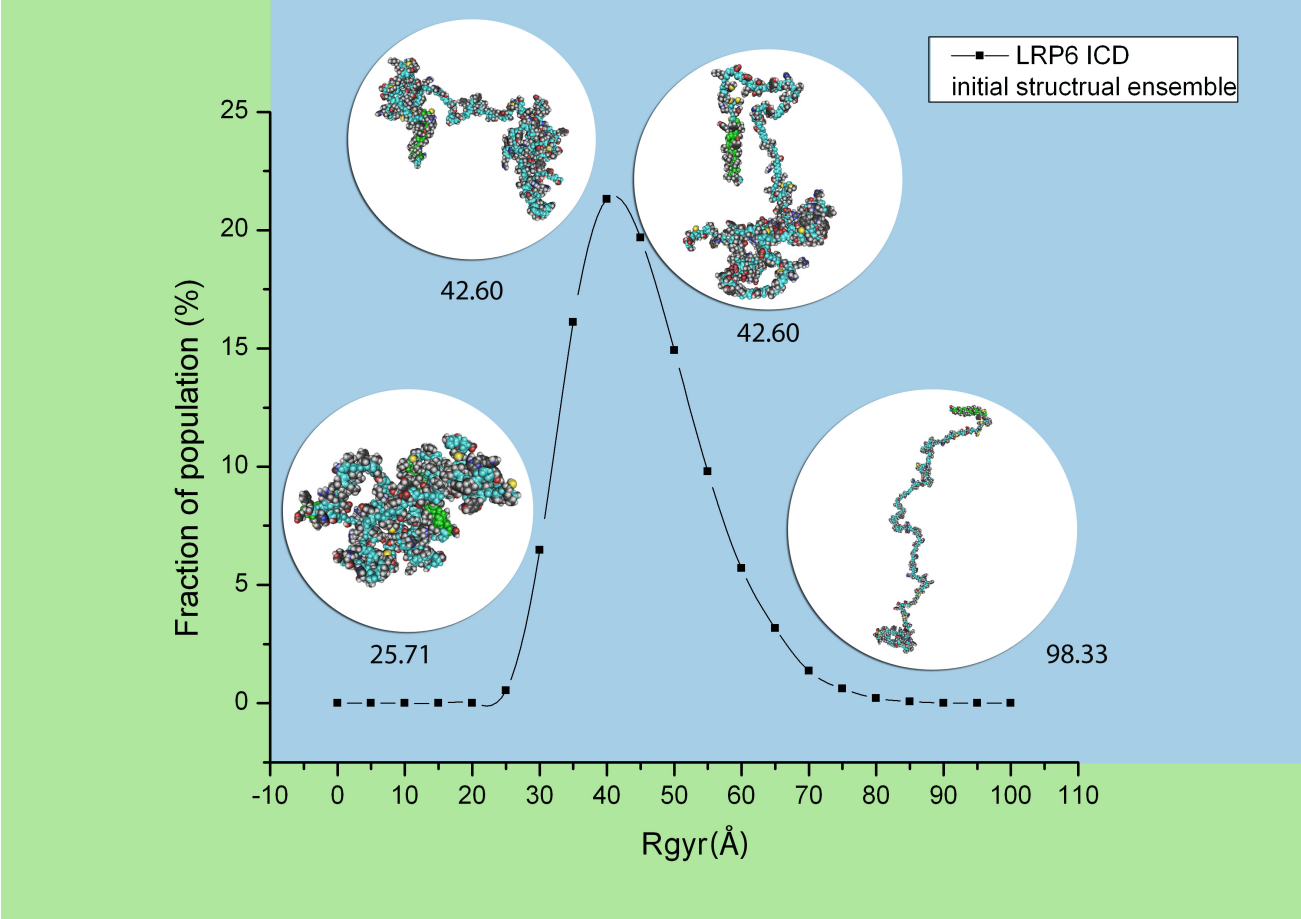
**Rgyr distribution of the initial conformational ensemble before filtration.** Conformers shown in the graph are some examples in the initial conformational ensemble of LRP6 intracellular domain. The number below each conformer is the value of its radius of gyration. A conformer with a smaller value of radius of gyration has a compact conformation (the structure on the left). A conformer with a larger value of radius of gyration has an extended conformation (the structure on the right). Two conformers with a mean value of radius of gyration are shown in the middle.

**Figure 3:**
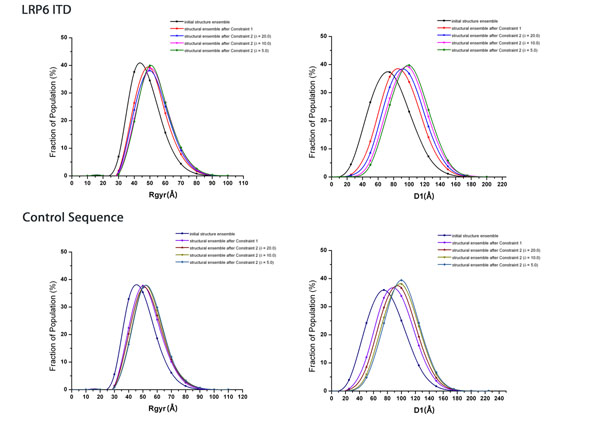
**Rgyr distributions and end-to-end distance distributions of D1 for LRP6 intracellular domain and control sequence.** The upper left graph displays the Rgyr distribution for LRP6 intracellular domain at δ set to 20.0Å, 10.0Å, and 5.0Å. The lower left graph displays the Rgyr distribution for control sequence at δ set to 20.0Å, 10.0Å, and 5.0Å. The upper right graph displays the D1 distribution for LRP6 intracellular domain at δ set to 20.0Å, 10.0Å, and 5.0Å. The lower right graph displays the D1 distribution for control sequence at δ set to 20.0Å, 10.0Å, and 5.0Å.

Radius of gyration (Rgyr) measures the openness of the whole structure. A structure with a larger Rgyr has more sparse atoms within it. Figure 2 displays the Rgyr distribution of the initial conformational ensemble (before filtration) in the LRP6 intracellular domain simulation experiment. The number of generated conformers and average Rgyr are provided in the second column in Table 1. Conformers with different values of Rgyr were checked. It was observed that conformers with smaller Rgyr have more compact structures; while, conformers with larger Rgyr tend to adopt more open or extended conformations. In this distribution, the conformation of the generated conformers changes from compact, to more open, to more extended as their Rgyr increases. Simulation experiments were carried out both for the LRP6 intracellular domain (ICD) and the control sequence. See results in Table 1 and Table 2. A Unix script was written to obtain 10000 conformers that pass Constraint 1 and Constraint 2 (δ = 20.0Å) out of the initial structural ensemble. These 10000 conformers were then filtered by Constraint 2 with parameter δ set to 5.0Å and 10.0Å. This parameter represents the distance from the vertical plane to the plane defined by the transmembrane helix and origin point (0,0,0). The average Rgyr of the structural ensemble gets larger after each constraint is applied, as shown in Table 1, Table 2 and Figure 3. In both the LRP6 intracellular domain and control sequence simulation experiments, after each filtration, conformers in the structural ensemble surviving from the spatial constraints tend to possess more open or extended conformations. This was also indicated by the observation that after each constraint more fractions of structural ensemble appear to have Rgyr larger than the average (i.e. the Rgyr distribution curves of structural ensembles after Constraint 1 and Constraint 2 shift to the right of the Rgyr distribution curve of the initial structural ensemble). In addition, when the distance δ gets smaller, the average Rgyr in the structure ensemble that survived following Constraint 2 gets bigger.

**Table 1:** Rgyr simulation results for LRP6 intracellular domain.

LRP6 ICD simulation	Initial structural ensemble	Structural ensemble after constraint 1	Structural ensemble after constraint 2		
			
			δ=20.0	δ=10.0	δ=5.0
**No. Structures**	396339	36025	10000	4939	2192
**Average. Rgyr**	42.5726	46.4323	47.4313	48.3623	48.7313
**Minimum. Rgyr**	20.7308	23.1174	23.1174	26.1774	26.1774
**Maximum. Rgyr**	98.6045	95.0215	95.0215	95.0215	95.0215

**Table 2:** Rgyr simulation results for control sequence.

Control sequence simulation	Initial structural ensemble	Structural ensemble after constraint 1	Structural ensemble after constraint 2		
			
			δ=20.0	δ=10.0	δ=5.0
**No. structures**	181833	33556	10000	5632	3276
**Average. rgyr**	44.1536	47.9429	48.7239	49.6311	59.7834
**Minimum. rgyr**	20.4266	22.1955	23.8913	24.7448	27.1459
**Maximum. rgyr**	101.0442	101.0442	93.7720	93.7720	92.3932

The control sequence has LRP6 transmembrane region and poly(Pro-Ser)_50_ polypeptide substituting LRP6 intracellular domain. The theoretical maximum Rgyr for a completely extended chain (a polypeptide containing 100 Gly residues, i.e. poly(Gly)_100_ polypeptide) is calculated from TraDES to be around 90Å. The poly(Gly)_100_ polypeptide is constrained to take a β-strand conformation in its trajectory distribution file (Phi = -119; Psi = 113; Peak Magnitude = 100).

### End-to-end distance distribution

Five end-to-end distances with equal length were calculated in the LRP6 intracellular domain simulation experiment. Each distance contains at least one conserved PPP(S/T)PX(S/T) motif. See Table 3. For each end-to-end distance, the difference between the average Rgyr of the initial structural ensemble and that of the structural ensemble after Constraint 1 is provided in the column titled as “∆mean (Constraint1)”. The difference between the average Rgyr of the initial structural ensemble and that of the structure ensemble after Constraint 2 under different values of distance δ are shown in the columns under the title of “∆mean (Constraint2)”. For both the LRP6 intracellular domain and control sequence, out of the five end-to-end distances, the distribution of D1 displays largely increased mean values of the structural ensembles after each constraint. This was indicated by the positive differences in Table 3. It also proves that after each constraint the average value of D1 gets larger. In Figure 3, the D1 distribution curves of structural ensembles after Constraint 1 and after Constraint 2 move to the right of the D1 distribution curve of the initial structural ensemble. Meanwhile, more fractions of structural ensemble after each constraint are found at a larger value of D1 on the distribution curves. This indicates that the region corresponding to D1, within the LRP6 intracellular domain conformers in the surviving structural ensemble, prefers an elongated or extended conformation. This region starts right from the beginning of the LRP6 intracellular domain and extends to the end of the first PPP(S/T)PX(S/T) motif. It is the closest membrane region inside the LRP6 intracellular domain. Additionally, as the distance δ gets smaller, the mean of D1 of the structural ensemble after Constraint 2 also gets bigger. However, for both the LRP6 intracellular domain and control sequence, the rest four end-to-end distances’(D2-D5) distributions show no prominent changes compared to the change in D1 distribution after applying each constraint and furthermore they overlap with each other as seen in Figure S2 (in the Additional File 2).

**Table 3:** End-to-end distance simulation results for LRP6 intracellular domain.

	Length	Motifs contained	start-end	∆mean (Constraint1)	∆mean (Constraint2)		
					
					δ=20	δ=10	δ=5
**D1**	103aa	S/T cluster; Motif 1	24-126	12.7220	17.5067	21.9135	24.2415
**D2**	103aa	Motif 1&2	64-166	0.8405	1.9484	2.4047	2.7648
**D3**	103aa	Motif 1,2&3	106-208	-1.3586	-2.7764	-2.8862	-3.0856
**D4**	103aa	Motif 2,3&4	124-226	-1.6533	-3.5066	-3.4477	-3.5721
**D5**	103aa	Motif 2,3,4 &5	141-243	-1.4545	-3.1360	-3.1388	-3.3186

The table shows the length, motif contained, starting and ending positions in LRP6 simulation sequence, and the differences between the average Rgyr of structural ensembles after each constraint and the initial structural ensemble.

## Discussion

### LRP6 intracellular domain structure ensemble favors an elongation form when the Wnt/β-canonical pathway initiates

In the LRP6 intracellular domain simulation experiment, greater proportions of the structural ensemble after each spatial constraint are observed to have Rgyr of a larger value than the average in comparison with the initial structural ensemble (Figure 3). It shows that the two spatial constraints make the LRP6 intracellular domain likely to adopt a more open or elongated conformation. The plasma membrane and neighboring assemblies formed by Wnt-FZD-DVL-AXIN-GSK3 or neighboring LRP6 aggregation could limit the LRP6 intracellular domain to form fewer numbers of random coiled structures. Instead, the LRP6 intracellular domain tends to form more elongated conformations as the spatial constraints exclude its volume near the membrane in the cell. *In vivo*, plasma membrane and nearby assemblies or molecules could result in a natural elongation of the LRP6 intracellular domain when a Wnt signal triggers the pathway. Such elongation behavior might grant kinases CK1 and GSK3 open access to the phosphorylation sites within the LRP6 intracellular domain, and subsequently LRP6 could be activated through these phosphorylation events. We propose that when the Wnt pathway initiates, the LRP6 intracellular domain is elongated to reduce the auto-inhibition before it is activated.

A conformational change occurs to the LRP6 intracellular domain structural ensemble after applying spatial constraints. It is intriguing to investigate if any conformational change could occur in the subsequences within the LRP6 intracellular domain during Wnt canonical pathway initiation. The distributions of the five calculated end-to-end distances could reflect the openness of the subsequences in the LRP6 intracellular domain. The first end-to-end distance D1, which measures the openness of the region that is closest to the membrane on the LRP6 intracellular domain. The distribution curves for D1 show that this region gets longer in more conformers out of the structural ensemble after filtration (Figure 3). This implies that this near-membrane region in the LRP6 intracellular domain elongates or extends when its conformational space is limited by the plasma membrane and nearby assemblies or molecules. Such an extended conformation could allow CK1 to more easily reach the S/T cluster and initiate phosphorylation. This may also explain the experimental finding that S/T cluster phosphorylation by CK1 promotes the downward activation of the PPP(S/T)PX(S/T) motif [8,23,24]. On the contrary, the end-to-end distances D2, D3, D4, and D5 hardly show any changes in their distribution curves between original and filtered structural ensembles (See Figure S2). The means of these distributions of structural ensemble after filtration are in fact smaller than that of the initial structural ensemble. This suggests the regions corresponding to these distances are on average less extended in the conformers surviving from filtration. The protein regions corresponding to the five end-to-end distances are gradually further away from the transmembrane helix, which determines the location of the plasma membrane. The region corresponding to D1 is the closest to the plasma membrane followed by D2. The observations on the distribution curves of these distances suggest that the spatial constraints exclude to a great extent the volume of the LRP6 intracellular domain at the near-membrane location in the cell.

Additionally, the same behaviors are observed in Rgyr distributions and end-to-end distributions in the simulation experiment as for the control sequence. We observed structural changes that can be demonstrated in a hypothetical sequence with as much as 50% proline and 50% serine. Such changes could function as a mechanism by which high rates of mutations could be tolerated whilst conserving function. Hence, it can be concluded that such an elongation process induced by membrane and neighboring assembly/aggregation is sequence independent but maybe compositional dependent.

### Effects of the two spatial constraints

Near-membrane serves as the key point in the simulation study. The membrane-anchor issue has been discussed in several published papers that claimed the LRP6 intracellular domain needs anchoring to the membrane to process signaling [15]. Arrow/LRP5/LRP6 mutants without the extracellular domain with which to anchor to the membrane constitutively activate the β-catenin pathway in mammalian cells. The LRP5 intracellular domain is unable to activate the signaling pathway unless it is anchored to the membrane [25-27]. In the simulation, the horizontal plane mimics the constraint imposed by the membrane plane. The vertical plane mimics the constraint imposed by nearby assemblies or molecules. Experiments show that the components in the assembly, for example, DVL, AXIN and GSK3 accumulate near the membrane when Wnt interacts with FZD and initiates the pathway [7]. Furthermore, CK1 that is responsible for the S/T cluster phosphorylation events is a near-membrane kinase [23,37]. The second constraint also occurs near the membrane. If the second constraint is more stringent and the vertical plane gets closer to the conformer (i.e.δ gets smaller), the spatial volume of the conformer is excluded more. Such an excluded volume effect forces the conformer of the LRP6 intracellular domain to go through an elongation process. We propose this elongation might be necessary for the phosphorylation of the LRP6 intracellular domain. Liu and colleagues [27] demonstrated that a truncated LRP6 comprising of its transmembrane and cytoplasmic domains is expressed as a constitutively active monomer whose signaling ability is inhibited by forced dimerization. Also, Wnts are shown to activate canonical signaling through LRP6 by inducing an intracellular conformational switch which relieves allosteric inhibition imposed on the intracellular domains. This paper published in 2003 is the only one until now on the conformational behaviour of the LRP6 intracellular domain through experiments. There is however no evidence to prove such a conformational switch in terms of indicating the changes in the LRP6 intracellular domain structural ensemble. In the paper published by Yasui *et al*. [38], the authors conclude that the LRP6 extracellular domain does not form homodimers in solution and speculate that weak dimerization may occur only at the cell surface where the receptors are confined in the 2D plane. In our current simulation study, we focus on the conformational change of LRP6 intracellular domain under spatial constraints in the initiation of the canonical Wnt signalling pathway. Our results show that the spatial constraints cause the structural ensemble of the intracellular domain to adopt an extended or elongated form which relieves the allosteric inhibition. This provides another explanation for why wild-type LRP6 and LRP6 mutant without an extracellular domain behave differently with or without the presence of Wnt. The LRP6 mutant without an extracellular domain is free from the auto-inhibitory effect imposed by its extracellular domain. The LRP6 intracellular domain anchored to the plasma membrane only faces the spatial constraint caused by the plasma membrane. It can adopt a more open or elongated conformation to relieve the auto-inhibition caused by this unfolded region itself, to allow CK1 and GSK3 access. For wild-type LRP6, without the presence of Wnt, membrane constraint is not enough to relieve the auto-inhibition caused by its extracellular and intracellular domains. It requires another constraint to relieve the auto-inhibitory effect caused by the extracellular domain. When Wnt is present, it forms a complex with FZD and interacts with the LRP6 extracellular domain. Though this interaction may be weak, the conformational space of the LRP6 intracellular domain is excluded. The domain is therefore forced to adopt a more open or extended structure for it to reduce the tension. Wnt-FZD hence imposes another spatial constraint to LRP6. In the initiation complex, Wnt is not the only component; FZD, DVL, AXIN and GSK3 also participate in the process. Hence, they together could form the second spatial constraint on LRP6 to amplify the reduction of auto-inhibitory effect. Such amplification would be helpful to the activation of the LRP6 intracellular domain and the stabilization of β-catenin. Our model and results can help explain the results obtained by Liang *et al* who recently discovered that the previously functional unknown protein TMEM198 is able to promote LRP6 phosphorylation in the Wnt signalling pathway. TMEM198 functions as a membrane scaffold protein, assembling kinases and substrates into a higher-molecular-weight complex prior to phosphorylation, but it promotes LRP6 phosphorylation through a mechanism independent of FZD and DVL [39]. Like FZD, TMEM198 could recruit CK1 as well as other molecules to form a nearby molecular assembly close to LRP6. Any nearby assembly, together with the membrane, can impose the spatial constraints to the conformational space of the LRP6 intracellular domain so that this region will be elongated for kinases CK1 and GSK3 to gain easy access for phosphorylation. Liang *et al*. observe TMEM198 to associate with LRP6, however, unlike FZD, the interaction is likely mediated by the transmembrane domains between LRP6 and TMEM198 which can bring the TMEM198-CK1 complex more close to LRP6. The findings in Liang *et al*. also demonstrate that near-membrane is the key point in the simulation model. The interaction between TMEM198 and LRP6 at transmembrane domains takes place at the membrane plane. It amplifies the vertical spatial constraint by recruiting CK1 which is near-membrane localized. The spatial constraints can come from any nearby molecules or molecular assemblies. These include neighboring LRP6 molecules in the signalosome/aggregation model, Wnt-FZD-DVL-AXIN-GSK3 assembly in the sequential/amplification model or other discovered molecular assemblies such as the TMEM198-CK1 complex reported in the paper by Liang *et al*.

### Elongation makes the phosphorylation of unfolded protein regions easier

**Figure 4:**
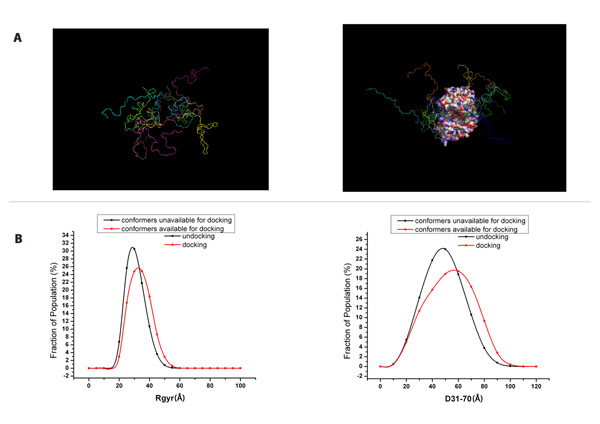
**Simulation results from the study on structure [PDB:1CMK]** A. Examples of the generated conformers of the constructed 100mer peptide. The conformers are aligned to the peptide in [PDB:1CMK] complex. (Left): Conformers not available for docking. The regions near binding site appear to be more random coiled. For a conformer unavailable for docking, the root mean squared distance (RMSD) of the alignment between the binding peptide and the binding region in the constructed peptide is bigger than the threshold 0.5. (Right): Conformers available for docking. B. Rgyr distribution and end-to-end distance distribution for D31-70 for the constructed 100mer peptide. The left graph displays the Rgyr distribution for the constructed 100mer peptide. The right graph displays the end-to-end distribution of D31-70 for the constructed 100mer peptide.

We proposed that the elongation form may be required for the phosphorylation of the LRP6 intracellular domain. Since there is no structure for the LRP6 intracellular domain present in the structural databases, we used a structure complex [PDB:1CMK] to demonstrate that in a general case, elongation is required for phosphorylation taking place in the conformation of an unfolded protein region. [PDB:1CMK] contains a cAMP dependent protein kinase catalytic subunit and its inhibitor, a 22aa long peptide binding to the kinase catalytic site. The simulation procedure is provided in the Additional File 3. We constructed a 100mer sequence that includes the binding peptide in the middle and a repeated proline-serine extending to both terminals. We used TraDES to generate a structural ensemble with the constructed sequence. Out of the 347426 conformers generated, 10000 passed the aligning, merging and crashes-checking requirements. These survival conformers are available for docking. Figure 4A shows some examples of the conformers that are available and unavailable for docking. We calculated the Rgyr and end-to-end distances of Region 1-40, Region 31-70, Region 61-100 along the sequence and compared the distribution curves between 10000 conformers that are available for docking and 347426 conformers that are not available for docking (Figure 4B and Figure S3 in the Additional File 4). We used a t-test to see whether there is a big difference between the means of the datasets (Table 4). For Rgyr and end-to-end distance of the three regions, the p-values are significantly small (p<2.2e-16). This indicates that the structures available for the kinase to access and bind on average have larger Rgyr and longer end-to-end distances. We can therefore say that the structural ensemble available for docking becomes more open and elongated for kinases to access through. This proposition fits as elongation can reduce the auto-inhibition of the unfolded protein’s random coiled structure. In general, the elongation form induces the phosphorylation sites to become more exposed so that kinases may easily interact.

**Table 4:** T-test results on the constructed 100mer peptide.

t test			
**Alternative hypothesis:** **True difference in means is less than 0, i.e. the mean of undocking conformers’ Rgyr or end-to-end distance is less than that of docking conformers**			

	**Mean of undocking conformers**	**Mean of docking conformers**	**p-value**

**Rgyr**	28.4383	31.4704	<2.2e-16
**D1-40**	40.0255	41.8216	<2.2e-16
**D31-70**	43.6516	48.8396	<2.2e-16
**D61-100**	40.2149	41.3728	1.032e-14

The table shows t-test results on the Rgyr distributions and end-to-end distance distributions of docking and undocking structural ensembles of the constructed 100mer peptide.

## Conclusions

We compared the Rgyr distributions of structure ensembles of the LRP6 intracellular domain before and after applying spatial constraints. We observe that the whole structure becomes open or extended and find that the near-membrane region appears to be elongated with given horizontal and vertical spatial constraints. During the initiation, the spatial constraints caused by the plasma membrane and nearby assemblies or molecules force an elongation form to dominate the conformational space of the LRP6 intracellular domain. We demonstrated that such an elongation process is required for unfolded protein structures because it can relieve the auto-inhibitory effect and grant kinases easy access. The near-membrane LRP6 intracellular domain extension could expose the S/T cluster phosphorylation site for CK1, which subsequently promotes downward PPP(S/T)PX(S/T) phosphorylation events. This study elaborates details on the activation of LRP6 through its conformational change in the current Wnt/β-catenin pathway initiation models. TraDES provides a new way to investigate signal transduction mechanisms through computational simulations and bioinformatics methods. More importantly, it demonstrates a way to study the conformational behavior of other proline-rich unfolded protein regions including those in signaling pathways and mechanobiological systems. The Wnt/β-catenin signaling pathway plays important roles in cancer and diseases. The LRP5/6 mutation is responsible for bone density disorders, ocular disorders and disorders of cholesterol and glucose metabolism. The findings in this study could pave the way to the development of new therapeutics through structure based drug design with the consideration of spatial constraints imposed by cellular components. Experiments proposed to validate the LRP6 elongation model are single-molecule fluorescence resonance energy transfer (SM-FRET) and time-resolved fluorescence resonance energy transfer (TR-FRET). TraDES was originally validated with successful comparison to TR-FRET distribution [30]. SM-FRET and TR-FRET have been applied to study the conformations of full-length p53, which has both folded and intrinsically disordered domains [40]. SM-FRET can measure the radius of gyration of the LRP6 intracellular domain. TR-FRET can measure the end-to-end distance distribution within the LRP6 intracellular domain. Collectively these experiments will provide significant validation of the findings presented in this study.

## Methods

### Generation of conformers of LRP6 intracellular domain

The conformers of the LRP6 intracellular domain were generated using programs VISTRAJ and FOLDTRAJ from TraDES package [30], by providing the corresponding segment sequence. The sequence used was the 1613-residue LRP6 precursor retrieved from Uniprot database with definition line:

>sp|O75581|LRP6_HUMAN Low-density lipoprotein receptor-related protein 6 OS=Homo sapiens GN=LRP6 PE=1 SV=1

**Figure 5:**
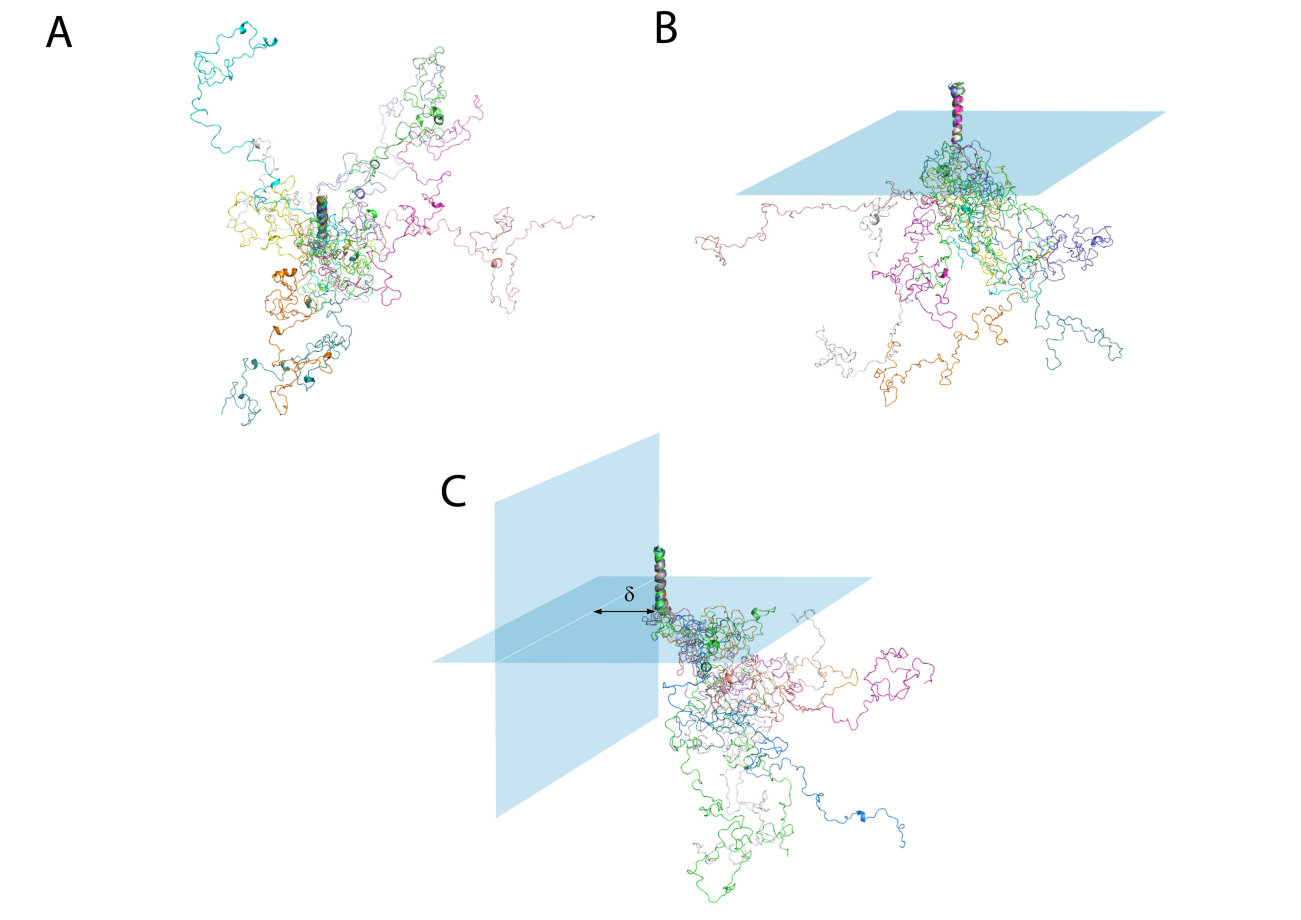
**Illustration of the spatial constraints.** A. Ten aligned conformers of LRP6 intracellular domain which are not filtered by any spatial constraints. They are free to explore a spherical shaped conformational space. B. Constraint 1: Horizontal Plane Ten aligned conformers of LRP6 intracellular domain which pass the horizontal spatial constraint, i.e. membrane plane. They can explore a hemispherical conformational space. Signal peptide and extracellular domain are deleted. The transmembrane region (23 residues) is constrained as an α-helix. C. Constraint 2: Vertical Plane Ten aligned conformers of LRP6 intracellular domain which pass both horizontal and vertical spatial constraints. They are free to explore a smaller transected hemispherical shaped conformational space. The vertical constraint is imposed by the Wnt-FZD-DVL-AXIN-GSK3 assembly. δ is the distance between the vertical plane and transmembrane helix.

The 19-residue signal peptide region was deleted from the N-terminal. As the extracellular domain from residue 20 to residue 1370 has its structure derived from X-ray diffraction in PDB database with accession ID “1N7D” [PDB:1N7D], this region was also deleted. The regions containing transmembrane and intracellular domain were taken as the segment sequence (residue 1371 to residue 1613) to generate conformers. The segment sequence was used in VISTRAJ to generate a trajectory distribution file for LRP6 intracellular domain, which contains the probabilistic distribution of φ / ψ angles in Ramachandran space for each residue in the segment sequence. This segment is predicted to be unfolded and has no apparent secondary structure. The “standard” method was used with no secondary structure predictions added. In this way the trajectory generated for each amino acid residue was based on its observed distribution of φ / ψ angles in a non-redundant subset of the PDB database. The trajectory distribution file was then manually edited to constrain the 23 residues from N-terminal side (T1371-I1393) to take an α-helix conformation by replacing their random trajectory distribution with a helical distribution (Phi = -57°; Psi = -47°; Peak Magnitude =100). This was used to do the first filtration of the structural ensemble. The modified trajectory distribution file was next used by FOLDTRAJ to generate all-atom structure models of LRP6 segment sequence. FOLDTRAJ generates off-lattice unbounded all-atom protein structures by amino acid residue random walks. The φ / ψ angles of the residues are obtained by sampling the Ramachandran space based on the trajectory distribution, and side chain rotamers are sampled from backbone dependent rotamer frequencies. Figure 5A displays examples of generated conformers of LRP6 intracellular domain.

### Filtration of structural ensemble of LRP6 intracellular domain

The conformers generated have no geometrical boundaries other than the steric hindrance of the sequence itself. However, *in vivo*, LRP6 is a transmembrane receptor and its intracellular domain would have its available conformational space limited by the cell membrane. If a structure generated has part of the peptide penetrating the plasma membrane, the conformation is not feasible *in vivo*, so we exclude it from the structure ensemble through a defined horizontal plane mimicking the plasma membrane plane. During Wnt signaling pathway initiation, the assembly Wnt-FZD-DVL-AXIN-GSK3 or neighboring LRP6 molecule gets to the proximity of a LRP6 molecule. This will cause a second steric constraint to LRP6 intracellular domain. We defined a vertical plane perpendicular to the plasma membrane plane in order to further filter the structure ensemble. Figure 5 shows some conformers of LRP6 intracellular domain that pass only Constraint 1 (Figure 5B) or both Constraint 1 and Constraint 2 (Figure 5C).

#### Constraint 1: horizontal plane

In order to filter out the conformations that having part outside cell membrane, a program was developed to test whether a generated structure is in a conformation that can be bounded by a membrane. This check is done by constructing a virtual plane at the trans-membrane site (Residue I1393), which is perpendicular to the inner membrane helix region. The rest of residues that should be inside the membrane are checked for whether they lie on the opposite site of the plane of the inner membrane helix. A unix shell script was written to filter ensembles of structures that pass this constraint test in batches.

#### Constraint 2: vertical plane

To further filter the structural ensemble and simulate the situation when an assembly or a neighboring molecule gets close to LRP6, a program was developed to test if a conformation is bounded by a plane that is between the intracellular domain of LRP6 and a neighboring object. We built another virtual plane at a distance δ of 5.0Å, 10.0Å and 20.0Å to the trans-membrane helix. All the residues should locate on one side of this plane. Another unix shell script was written to complete this constraint based filtering.

### Measurement

Under each constraint, we measured the Radius of Gyration (Rgyr) to see the openness of the whole structure. In the meantime, the end-to-end distances with equal residues were measured to see the openness of LRP6 intracellular subsequences containing conserved PPP(S/T)PX(S/T) motifs. We can determine whether there is a conformational change by comparing the distributions of Rgyr end-to-end distances of the structural ensembles under each constraint.

#### Measurement of radius of gyration

The generated structures by TraDES package are stored in NCBI ASN.1 format. It contains the locations of all the atoms inside the structure including hydrogen. Thus the distances between atoms and the radius of gyration could be calculated directly from the locations of the atoms. Radius of gyration is a measure of the dimensions of the peptide chains in polymer physics. It is defined as follows:

Where *r_k_* is the position of individual atoms of the structure and *r_mean_* is the mean position of the atoms (defined as the center of gravity of the structure). Radius of gyration is a root mean square distance of individual atom to the center of the structure. The higher the radius of gyration is, the sparser the atoms are in the structure. Therefore, this term can measure the openness of the whole structure.

#### Measurement of end-to-end distance

The end-to-end distance is defined by the distance between the α-carbon of the residues with a number of residues in between. The exact distances are those between C24 and S126, G64 and V166, T106 and L208, E124 and S226, T141 and S243. The averages of the end-to-end distances over large population indicate the openness of the subsequence within LRP6 intracellular domain.

### The Rgyr distribution and end-to-end distance distribution

The Rgyr distributions (fraction of population in structural ensemble vs Rgyr) after Constraint 1 and Constraint 2 are plotted to compare the openness of structure ensemble. If mean of Rgyr has a shift to a higher value, it would indicate that the structure prefers an open and extended conformation based on the application of physical constraint. The end-to-end distance distributions after Constraint 1 and Constraint 2 are also plotted to compare the openness of the LRP6 intracellular domain subsequence. If average end-to-end distance turns larger, it indicates the subsequence within LRP6 intracellular domain favors an open and elongated form.

### Control experiment

A control sequence with the same length of LRP6 segment containing a transmembrane and intracellular domain was constructed. The control sequence has LRP6 transmembrane region and repeated proline-serine peptides substituting LRP6 intracellular domain. Conformers were generated using TraDES constraining transmembrane portion to adopt an alpha helix. This initial structural ensemble was then filtered by Constraint 1 and Constraint 2. Rgyr and end-to-end distances were calculated and plotted into distribution curves. The control sequence is the following. TNTVGSVIGVIVTIFVSGTVYFIPSPSPSPSPSPSPSPSPSPSPSPSSPSPSPSPSPSPSPS PSPSPSPSPSPSPSPSPSPSPSPSPSPSPSPSPSPSPSPSPSPSPSPSPSPSPSPSPSPSPSPS PSPSPSPSPSPSPSPSPSPSPSPSPSPSPSPSPSPSPSPSPSPSPSPSPSPSPSPSPSPSPSPS PSPSPSPSPSPSPSPSPSPSPSPSPSPSPSPSPSPSPSPSPSPSPSPSPSPS

### Program development

All the programs developed for Constraint 1 and Constraint 2 are based on NCBI C toolkit, MMDBAPI and libraries in TraDES package (http://trades.blueprint.org/). MMDBAPI implements data structures for describing biological sequence and 3D structure data and tools for easy access and manipulation of data, either in file system or in memory, based on ASN.1 standard. The TraDES package contains powerful function to manipulate and analyze the 3D structure of proteins such as aligning proteins by SVD (Singular Value Decomposition) method and to calculate Rgyr of structures. Shell scripts are created for processing and analyzing the structure ensemble in batch. The file format for storing 3D structures is .val file that can be visualized by Cn3D program from NCBI, or converted into PDB format. The whole simulation process can be viewed in the flow chart (Figure S4 in the Additional File 5).

## Competing interests

The authors declare that they have no competing interests.

## Authors' contributions

CL carried out the simulation studies, developed the software, performed the statistical analysis and drafted the manuscript. MY participated in the program development. CWVH participated in the design of the study, the program development, coordination and helped to draft the manuscript. All authors read and approved the final manuscript.

## Supplementary Material

Additional File 1 **Figure S1 Analysis of the human LRP6 protein [Swiss-Prot:O75581] using different predictors** The graphical output of each method and the corresponding interpretation is shown. The precise boundaries of ordered and disordered regions were derived from the corresponding text output (not shown). The intracellular domain is unfolded, whereas the extracellular domain is folded/ structured. Click here for file

Additional File 2 **Figure S2 End-to-end distance distributions of D2 to D5 for LRP6 intracellular domain and control sequence** The upper panel displays the D2 to D5 distributions for LRP6 intracellular domain at δ set to 20.0Å, 10.0Å, and 5.0Å.The lower panel displays the D2 to D5 distributions for control sequence at δ set to 20.0Å, 10.0Å, and 5.0Å. Click here for file

Additional File 3 Simulation procedure to obtain the constructed peptide’s structural ensemble that is available for docking to the catalytic site in structure [PDB:1CMK] Click here for file

Additional File 4 **Figure S3 End-to-end distance distributions of D1-40 and D61-100 for the constructed 100mer peptide** The two graphs display the end-to-end distributions of D1-40 and D61-100 for the constructed 100mer peptide. Click here for file

Additional File 5 Figure S4 Flow chart of the simulation process on LRP6 intracellular domain Click here for file

## References

[B1] Logan CY Nusse R The Wnt signaling pathway in development and disease Annual review of cell and developmental biology 2004 20 781 810 10.1146/annurev.cellbio.20.010403.113126 15473860

[B2] Clevers H Wnt/beta-catenin signaling in development and disease Cell 2006 127 3 469 480 10.1016/j.cell.2006.10.018 17081971

[B3] Klaus A Birchmeier W Wnt signalling and its impact on development and cancer Nature reviews Cancer 2008 8 5 387 398 10.1038/nrc2389 18432252

[B4] Wu D Pan W GSK3: a multifaceted kinase in Wnt signaling Trends in biochemical sciences 2010 35 3 161 168 10.1016/j.tibs.2009.10.002 19884009 PMC2834833

[B5] Angers S Moon RT Proximal events in Wnt signal transduction Nature reviews Molecular cell biology 2009 10 7 468 477 19536106 10.1038/nrm2717

[B6] Verheyen EM Gottardi CJ Regulation of Wnt/beta-catenin signaling by protein kinases Developmental dynamics : an official publication of the American Association of Anatomists 2010 239 1 34 44 10.1002/dvdy.22019 PMC3173947 19623618

[B7] Zeng X Huang H Tamai K Zhang X Harada Y Yokota C Almeida K Wang J Doble B Woodgett J Initiation of Wnt signaling: control of Wnt coreceptor Lrp6 phosphorylation/activation via frizzled, dishevelled and axin functions Development (Cambridge, England) 2008 135 2 367 375 10.1242/dev.013540 PMC5328672 18077588

[B8] Bilic J Huang Y-L Davidson G Zimmermann T Cruciat C-M Bienz M Niehrs C Wnt induces LRP6 signalosomes and promotes dishevelled-dependent LRP6 phosphorylation Science (New York, NY) 2007 316 5831 1619 1622 10.1126/science.1137065 17569865

[B9] Kimelman D Xu W beta-catenin destruction complex: insights and questions from a structural perspective Oncogene 2006 25 57 7482 7491 10.1038/sj.onc.1210055 17143292

[B10] Ha N-C Tonozuka T Stamos JL Choi H-J Weis WI Mechanism of phosphorylation-dependent binding of APC to beta-catenin and its role in beta-catenin degradation Molecular cell 2004 15 4 511 521 10.1016/j.molcel.2004.08.010 15327768

[B11] Liu C Li Y Semenov M Han C Baeg GH Tan Y Zhang Z Lin X He X Control of beta-catenin phosphorylation/degradation by a dual-kinase mechanism Cell 2002 108 6 837 847 10.1016/S0092-8674(02)00685-2 11955436

[B12] van Noort M Meeldijk J van der Zee R Destree O Clevers H Wnt signaling controls the phosphorylation status of beta-catenin The Journal of biological chemistry 2002 277 20 17901 17905 10.1074/jbc.M111635200 11834740

[B13] Molenaar M van de Wetering M Oosterwegel M Peterson-Maduro J Godsave S Korinek V Roose J Destrée O Clevers H XTcf-3 transcription factor mediates beta-catenin-induced axis formation in Xenopus embryos Cell 1996 86 3 391 399 10.1016/S0092-8674(00)80112-9 8756721

[B14] Brown SD Twells RC Hey PJ Cox RD Levy ER Soderman AR Metzker ML Caskey CT Todd JA Hess JF Isolation and characterization of LRP6, a novel member of the low density lipoprotein receptor gene family Biochemical and biophysical research communications 1998 248 3 879 888 10.1006/bbrc.1998.9061 9704021

[B15] He X Semenov M Tamai K Zeng X LDL receptor-related proteins 5 and 6 in Wnt/beta-catenin signaling: arrows point the way Development (Cambridge, England) 2004 131 8 1663 1677 10.1242/dev.01117 15084453

[B16] Springer TA An extracellular beta-propeller module predicted in lipoprotein and scavenger receptors, tyrosine kinases, epidermal growth factor precursor, and extracellular matrix components Journal of molecular biology 1998 283 4 837 862 10.1006/jmbi.1998.2115 9790844

[B17] Jeon H Meng W Takagi J Eck MJ Springer TA Blacklow SC Implications for familial hypercholesterolemia from the structure of the LDL receptor YWTD-EGF domain pair Nature structural biology 2001 8 6 499 504 10.1038/88556 11373616

[B18] Tamai K Semenov M Kato Y Spokony R Liu C Katsuyama Y Hess F Saint-Jeannet JP He X LDL-receptor-related proteins in Wnt signal transduction Nature 2000 407 6803 530 535 10.1038/35035117 11029007

[B19] Semënov MV Tamai K Brott BK Kühl M Sokol S He X Head inducer Dickkopf-1 is a ligand for Wnt coreceptor LRP6 Current biology : CB 2001 11 12 951 961 10.1016/S0960-9822(01)00290-1 11448771

[B20] Brennan K Gonzalez-Sancho JM Castelo-Soccio LA Howe LR Brown AMC Truncated mutants of the putative Wnt receptor LRP6/Arrow can stabilize beta-catenin independently of Frizzled proteins Oncogene 2004 23 28 4873 4884 10.1038/sj.onc.1207642 15064719 PMC2494703

[B21] Niehrs C Shen J Regulation of Lrp6 phosphorylation Cellular and molecular life sciences : CMLS 2010 67 15 2551 2562 10.1007/s00018-010-0329-3 20229235 PMC11115861

[B22] Zeng X Tamai K Doble B Li S Huang H Habas R Okamura H Woodgett J He X A dual-kinase mechanism for Wnt co-receptor phosphorylation and activation Nature 2005 438 7069 873 877 10.1038/nature04185 16341017 PMC2100418

[B23] Davidson G Wu W Shen J Bilic J Fenger U Stannek P Glinka A Niehrs C Casein kinase 1 gamma couples Wnt receptor activation to cytoplasmic signal transduction Nature 2005 438 7069 867 872 10.1038/nature04170 16341016

[B24] Yum S Lee S-J Piao S Xu Y Jung J Jung Y Oh S Lee J Park B-J Ha N-C The role of the Ser/Thr cluster in the phosphorylation of PPPSP motifs in Wnt coreceptors Biochemical and biophysical research communications 2009 381 3 345 349 10.1016/j.bbrc.2009.02.044 19309792

[B25] Mao B Wu W Li Y Hoppe D Stannek P Glinka A Niehrs C LDL-receptor-related protein 6 is a receptor for Dickkopf proteins Nature 2001 411 6835 321 325 10.1038/35077108 11357136

[B26] Mao J Wang J Liu B Pan W Farr GH Flynn C Yuan H Takada S Kimelman D Li L Low-density lipoprotein receptor-related protein-5 binds to Axin and regulates the canonical Wnt signaling pathway Molecular cell 2001 7 4 801 809 10.1016/S1097-2765(01)00224-6 11336703

[B27] Liu G Bafico A Harris VK Aaronson SA A novel mechanism for Wnt activation of canonical signaling through the LRP6 receptor Molecular and cellular biology 2003 23 16 5825 5835 10.1128/MCB.23.16.5825-5835.2003 12897152 PMC166321

[B28] Tamai K Zeng X Liu C Zhang X Harada Y Chang Z He X A mechanism for Wnt coreceptor activation Molecular cell 2004 13 1 149 156 10.1016/S1097-2765(03)00484-2 14731402

[B29] Piao S Lee S-H Kim H Yum S Stamos JL Xu Y Lee S-J Lee J Oh S Han J-K Direct inhibition of GSK3beta by the phosphorylated cytoplasmic domain of LRP6 in Wnt/beta-catenin signaling PloS one 2008 3 12 e4046 10.1371/journal.pone.0004046 19107203 PMC2603313

[B30] Feldman HJ Hogue CW A fast method to sample real protein conformational space Proteins 2000 39 2 112 131 10.1002/(SICI)1097-0134(20000501)39:2<112::AID-PROT2>3.0.CO;2-B 10737933

[B31] Yang ZR Thomson R McNeil P Esnouf RM RONN: the bio-basis function neural network technique applied to the detection of natively disordered regions in proteins Bioinformatics (Oxford, England) 2005 21 16 3369 3376 10.1093/bioinformatics/bti534 15947016

[B32] Dosztányi Z Csizmok V Tompa P Simon I IUPred: web server for the prediction of intrinsically unstructured regions of proteins based on estimated energy content Bioinformatics (Oxford, England) 2005 21 16 3433 3434 10.1093/bioinformatics/bti541 15955779

[B33] Linding R Russell RB Neduva V Gibson TJ GlobPlot: exploring protein sequences for globularity and disorder Nucleic acids research 2003 31 13 3701 3708 10.1093/nar/gkg519 12824398 PMC169197

[B34] Xue B Dunbrack RL Williams RW Dunker AK Uversky VN PONDR-FIT: a meta-predictor of intrinsically disordered amino acids Biochimica et biophysica acta 2010 1804 4 996 1010 20100603 10.1016/j.bbapap.2010.01.011 PMC2882806

[B35] Prilusky J Felder CE Zeev-Ben-Mordehai T Rydberg EH Man O Beckmann JS Silman I Sussman JL FoldIndex: a simple tool to predict whether a given protein sequence is intrinsically unfolded Bioinformatics (Oxford, England) 2005 21 16 3435 3438 10.1093/bioinformatics/bti537 15955783

[B36] Kim AS Kakalis LT Abdul-Manan N Liu GA Rosen MK Autoinhibition and activation mechanisms of the Wiskott-Aldrich syndrome protein Nature 2000 404 6774 151 158 10.1038/35004513 10724160

[B37] Price MA CKI, there's more than one: casein kinase I family members in Wnt and Hedgehog signaling Genes & development 2006 20 4 399 410 10.1101/gad.1394306 16481469

[B38] Yasui N Mihara E Nampo M Tamura-Kawakami K Unno H Matsumoto K Takagi J Detection of endogenous LRP6 expressed on human cells by monoclonal antibodies specific for the native conformation Journal of Immunological Methods 2010 352 (1-2) 153 160 19945460 10.1016/j.jim.2009.11.016

[B39] Liang J Fu Y Cruciat CM Jia S Wang Y Tong Z Tao Q Ingelfinger D Boutros M Meng A Niehrs C Wu W Transmembrane protein 198 promotes LRP6 phosphorylation and Wnt signaling activation Molecular and cellular biology 2011 31 13 2577 2590 10.1128/MCB.05103-11 21536646 PMC3133378

[B40] Huang F Rajagopalan S Settanni G Marsh RJ Armoogum DA Nicolaou N Bain AJ Lerner E Haas E Ying L Fersht AR Multiple conformations of full-length p53 detected with single-molecule fluorescence resonance PNAS 2009 106 49 20758 20763 10.1073/pnas.0909644106 19933326 PMC2791586

