# Supplementary material for: Near-membrane ensemble elongation in the proline-rich LRP6 intracellular domain may explain the mysterious initiation of the Wnt signaling pathway
Source: BMC Bioinformatics. 2011 Nov 30;12(Suppl 13):S13. doi: 10.1186/1471-2105-12-S13-S13 (PMC3278829; doi:10.1186/1471-2105-12-S13-S13)
Supplement: Additional File 1 — Figure S1 Analysis of the human LRP6 protein [Swiss-Prot:O75581] using different predictors The graphical output of each method and the corresponding interpretation is shown. The precise boundaries of ordered and disordered regions were derived from the corresponding text output (not shown). The intracellular domain is unfolded, whereas the extracellular domain is folded/ structured. [file 1471-2105-12-S13-S13-S1.pdf]

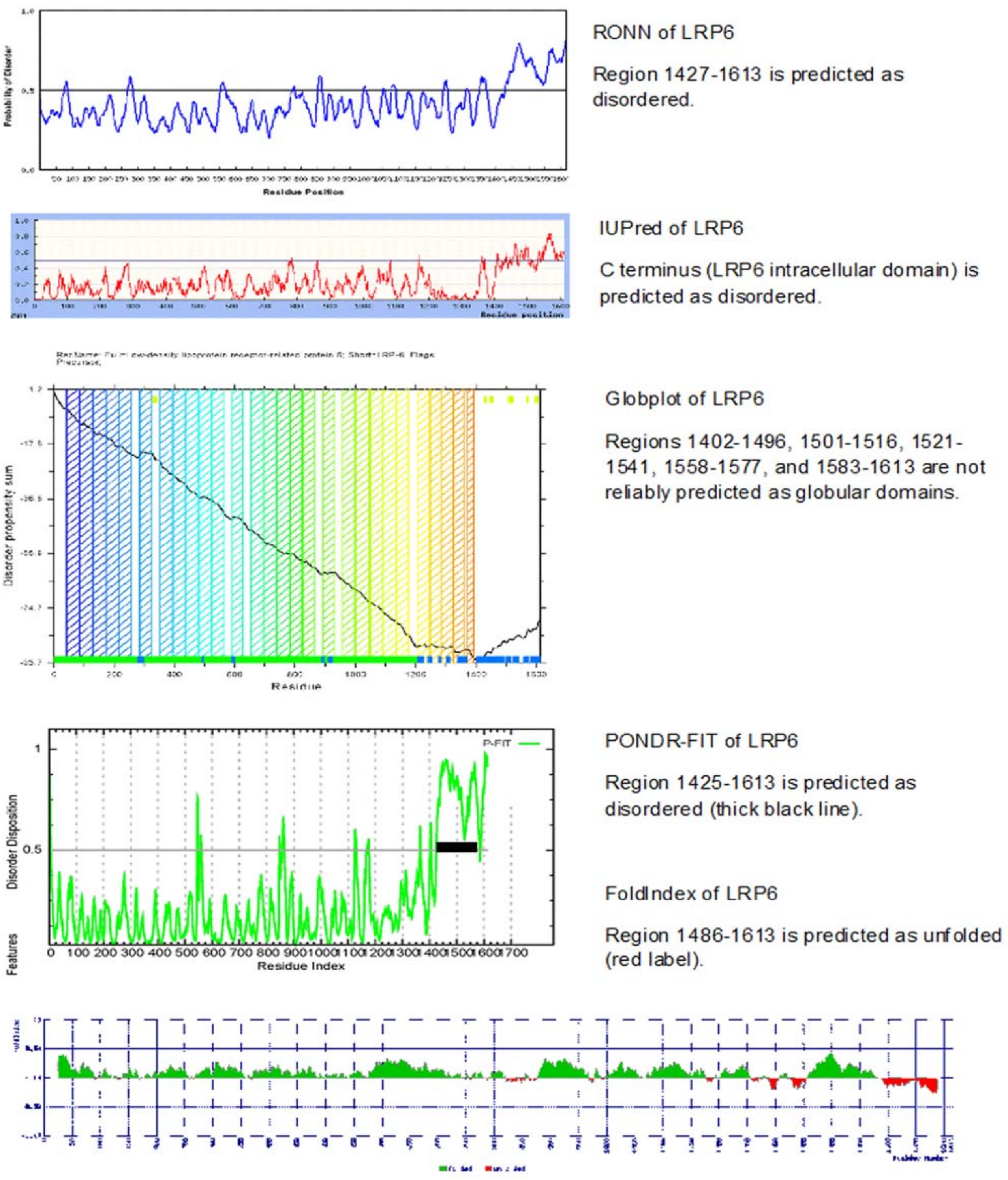

Figure S1- Analysis of the human LRP6 protein [Swiss-Prot:O75581] using different predictors.

The graphical output of each method and the corresponding interpretation is shown. The precise boundaries of ordered and disordered regions were derived from the corresponding text output (not shown). The intracellular domain is unfolded, whereas the extracellular domain is folded/ structured.
