# Supplementary material for: Near-membrane ensemble elongation in the proline-rich LRP6 intracellular domain may explain the mysterious initiation of the Wnt signaling pathway
Source: BMC Bioinformatics. 2011 Nov 30;12(Suppl 13):S13. doi: 10.1186/1471-2105-12-S13-S13 (PMC3278829; doi:10.1186/1471-2105-12-S13-S13)
Supplement: Additional File 2 — Figure S2 End-to-end distance distributions of D2 to D5 for LRP6 intracellular domain and control sequence The upper panel displays the D2 to D5 distributions for LRP6 intracellular domain at δ set to 20.0Å, 10.0Å, and 5.0Å.The lower panel displays the D2 to D5 distributions for control sequence at δ set to 20.0Å, 10.0Å, and 5.0Å. [file 1471-2105-12-S13-S13-S2.pdf]

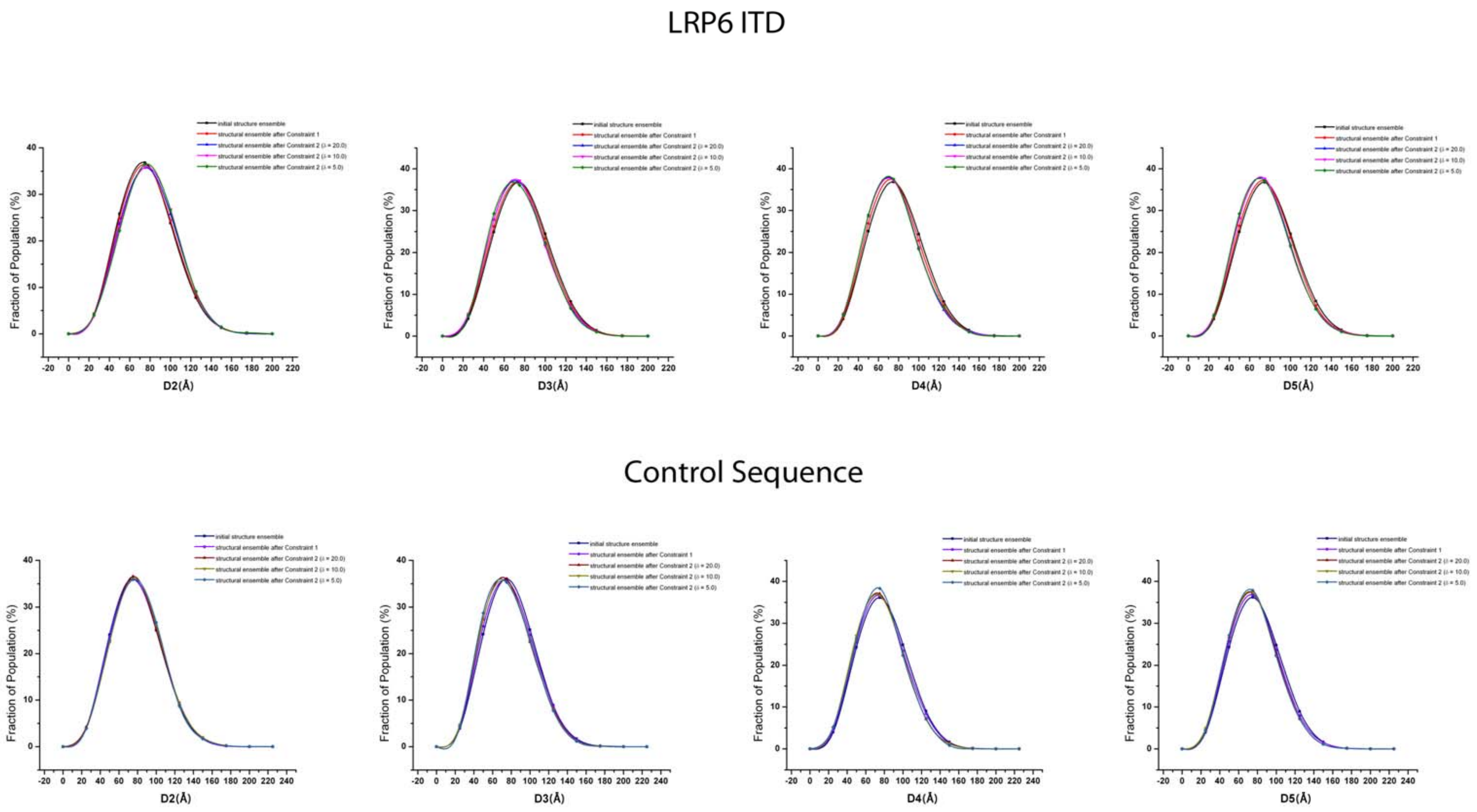

Figure S2-End-to-end distance distributions of D2 to D5 for LRP6 intracellular domain and control sequence.

The upper panel displays the D2 to D5 distributions for LRP6 intracellular domain at δ set to 20.0Å, 10.0Å, and 5.0Å.The lower panel displays the D2 to D5 distributions for control sequence at δ set to 20.0Å, 10.0Å, and 5.0Å.
