# Supplementary material for: Near-membrane ensemble elongation in the proline-rich LRP6 intracellular domain may explain the mysterious initiation of the Wnt signaling pathway
Source: BMC Bioinformatics. 2011 Nov 30;12(Suppl 13):S13. doi: 10.1186/1471-2105-12-S13-S13 (PMC3278829; doi:10.1186/1471-2105-12-S13-S13)
Supplement: Additional File 3 — Simulation procedure to obtain the constructed peptide’s structural ensemble that is available for docking to the catalytic site in structure [PDB:1CMK] [file 1471-2105-12-S13-S13-S3.pdf]

Simulation procedure to obtain the constructed peptide's structural ensemble that is available for docking to the catalytic site in structure [PDB:1CMK]

Alignment (SALIGN)

The structure [PDB:1CMK] chain I was aligned to the constructed 100mer peptide binding site by program SALIGN. SALIGN aligned two peptides (or part of the peptides) of the same length by superimposing them using a singular value decomposition (SVD) method. It takes in two structures and rotates and translates the second structure to align with the first one, then outputs the transferred structure to a new structure file. The exact parts of the sequence of the two sequences to be aligned can be specified respectively. There are three alignment methods – all atom, backbone and α-carbon. Each of the methods only takes their respective group of atoms into consideration. The backbone atoms were used for the 5 residues of the binding site in the constructed 100mer peptide. As a result of each alignment, the whole [PDB:1CMK] is transformed to the position and orientation where its chain E is superimposed with the binding site of the constructed 100mer peptide. The RMSD (root mean square deviation) of the alignment is calculated as a measure of quality of the alignment. A shell script was created to run this process in batch. The mean RMSD for conformers available for docking is 0.3463 and the best RMSD is 0.1660.

Merging (VALMERGE) and Crashes-checking (CRASHCHK)

After [PDB:1CMK] was aligned to the binding site of the constructed peptide, chain E of the [PDB:1CMK] structure was merged with the constructed 100mer peptide structure to form a protein complex. Then a check was done to determine whether the kinase catalytic subunit has steric crash with the constructed 100mer peptide. If no crashes were detected,

the binding site would be determined to be available for binding. Two programs VALMERGE and CRASHCHK were developed to do the task. VALMERGE could merge the structure in multiple files to form a complex. Individual chains could be specified so that only these are copied and merged into the output structure. CRASHCHK is a program that iteratively scans through each chain of the structure and output the number of atoms that are too close. The atom-atom distance is compared to the Van der Waal distances of the two atoms.

The developed programs in this study are included in TraDES package which can be found on the website http://trades.blueprint.org or downloaded from the ftp site ftp://ftp.blueprint.org/pub/TraDES.
