# Supplementary material for: Near-membrane ensemble elongation in the proline-rich LRP6 intracellular domain may explain the mysterious initiation of the Wnt signaling pathway
Source: BMC Bioinformatics. 2011 Nov 30;12(Suppl 13):S13. doi: 10.1186/1471-2105-12-S13-S13 (PMC3278829; doi:10.1186/1471-2105-12-S13-S13)
Supplement: Additional File 4 — Figure S3 End-to-end distance distributions of D1-40 and D61-100 for the constructed 100mer peptide The two graphs display the end-to-end distributions of D1-40 and D61-100 for the constructed 100mer peptide. [file 1471-2105-12-S13-S13-S4.pdf]

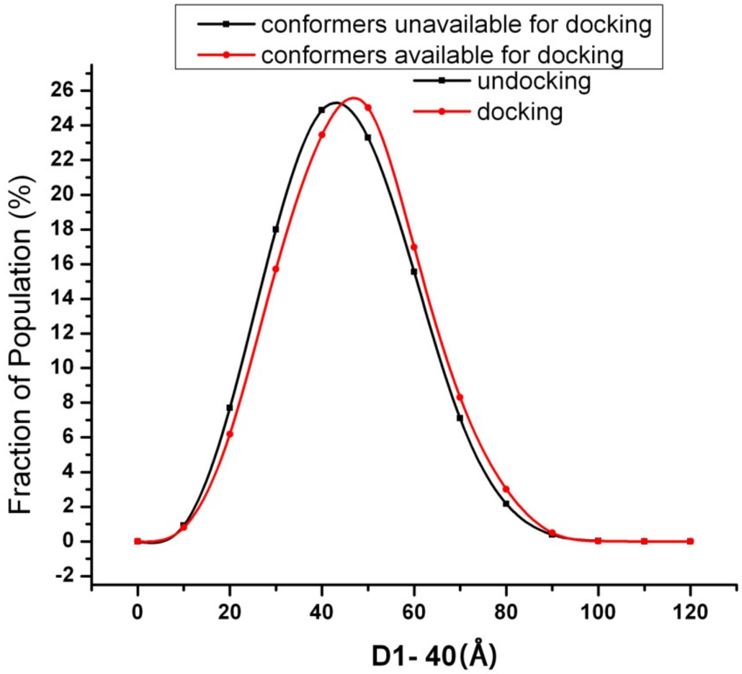

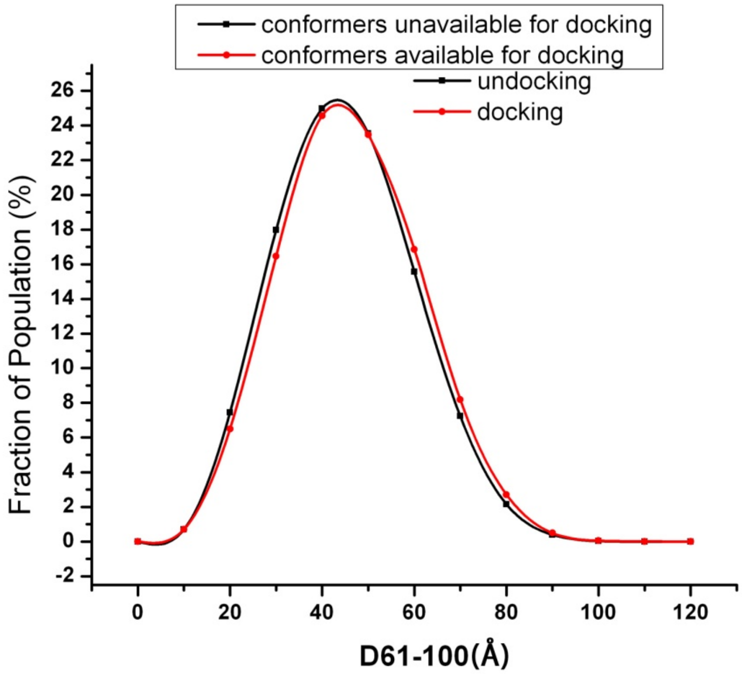

Figure S3-End-to-end distance distributions of D1-40 and D61-100 for the constructed 100mer peptide.

The two graphs display the end-to-end distributions of D1-40 and D61-100 for the constructed 100mer peptide.
