# Supplementary figures and images for: Near-membrane ensemble elongation in the proline-rich LRP6 intracellular domain may explain the mysterious initiation of the Wnt signaling pathway
Source: BMC Bioinformatics. 2011 Nov 30;12(Suppl 13):S13. doi: 10.1186/1471-2105-12-S13-S13 (PMC3278829; doi:10.1186/1471-2105-12-S13-S13)

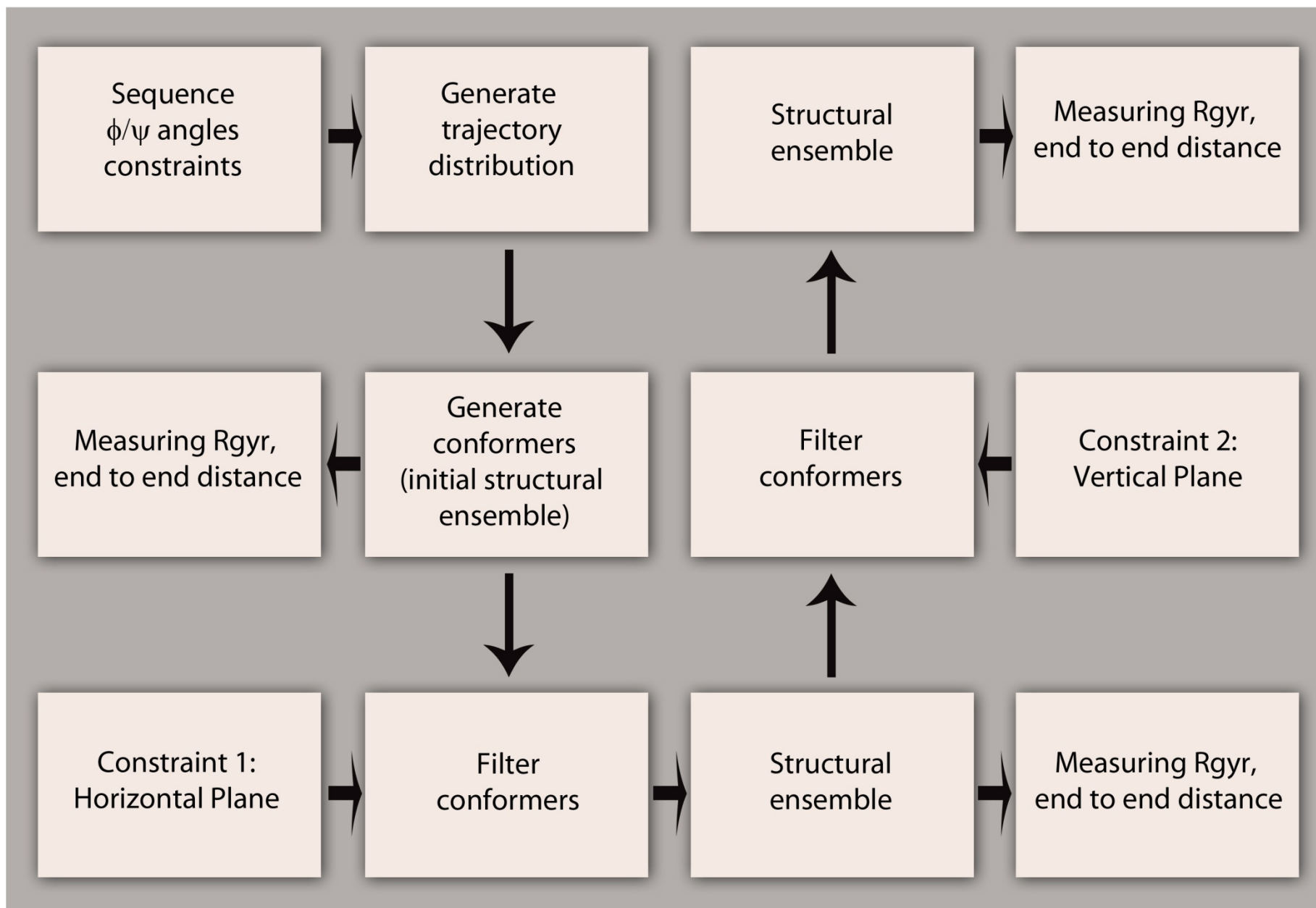

Figure S4-Flow chart of the simulation process on LRP6 intracellular domain.

Supplement: Additional File 5 — Figure S4 Flow chart of the simulation process on LRP6 intracellular domain [file 1471-2105-12-S13-S13-S5.pdf]
